# Association between Intratumoral CD8+ T Cells with FoxP3+ and CD163+ Cells: A Potential Immune Intrinsic Negative Feedback Mechanism for Acquired Immune Resistance

**DOI:** 10.3390/cancers14246208

**Published:** 2022-12-15

**Authors:** Sotirios P. Fortis, Michael Sofopoulos, Maria Goulielmaki, Niki Arnogiannaki, Alexandros Ardavanis, Sonia A. Perez, Angelos D. Gritzapis, Constantin N. Baxevanis

**Affiliations:** 1 Cancer Immunology and Immunotherapy Center, Cancer Research Center, Saint Savas Cancer Hospital, 11522 Athens, Greece; 2 Pathology Department, Saint Savas Cancer Hospital, 11522 Athens, Greece; 3 First Medical Oncology Clinic, Saint Savas Cancer Hospital, 11522 Athens, Greece

**Keywords:** tumor microenvironment, acquired immune resistance, CD8+ T cells, FoxP3+ cells, CD163+ cells, breast cancer

## Abstract

**Simple Summary:**

The cancer immunoediting theory states that tumor evolution is based on continuous and dynamic interactions between the immune system and tumor cells. Through these interactions, the endogenous antitumor immunity (EAI) is generated, which exerts a selective pressure to sculpt tumor phenotypes throughout all stages of tumor development. However, following chronic stimulation by tumor cells, immune lymphocytes express an “exhausted” phenotype and become anergic. Thus, therapies that reactivate the EAI (primarily immunotherapies) are most effective for inducing long-lasting clinical results. Nevertheless, the majority of patients relapse after an initial response to immunotherapies. The reason for this is that tumors develop acquired immune resistance (AIR) suppressing the EAI. In this study, we demonstrate that high frequencies of CD163+ and FoxP3+ cells in the tumors of patients with breast cancer co-exist with CD8+ cells only when the latter are also in dense frequencies. This type of AIR is associated with poor overall survival.

**Abstract:**

Acquired immune resistance (AIR) describes a situation in which cancer patients who initially responded clinically to immunotherapies, after a certain period of time, progress with their disease. Considering that AIR represents a feedback response of the tumor against the immune attack generated during the course of immunotherapies, it is conceivable that AIR may also occur before treatment initiation as a mechanism to escape endogenous adaptive antitumor immunity (EAAI). In the present study, we assessed the EAAI in paraffin-embedded breast primary tumor tissue samples and drew correlations with the clinical outcomes. In particular, we analyzed densities of CD8+ cells as elements mediating antitumor cytotoxicity, and of CD163+ and FoxP3+ cells as suppressor elements. We found a direct correlation between the densities of CD8+ cells and of CD163+ and/or FoxP3+ cells in the vast majority of patients’ tumors. Importantly, the vast majority of patients whose tumors were overpopulated by CD8+ cells developed AIR, which was characterized by high intratumoral CD163+ and/or FoxP3+ cell densities and reduced overall survival (OS). We also showed that AIR depends on the levels of CD8+ cell-ratios in the tumor center to the invasive margin. Our data suggest that tumors develop AIR only when under a robust endogenous immune pressure.

## 1. Introduction

The use of immunotherapies for fighting cancer has provided definite proof that T lymphocytes are capable of developing robust antitumor immunity, resulting in tumor cell destruction. However, despite impressive clinical responses with immunotherapies, the majority of patients either do not respond or relapse after initial responses. It is now increasingly recognized that, in most of these cases, tumors emerge when evading immune surveillance. Immune resistance mechanisms leading to immune escape possess a key role for tumor progression and therefore provide serious obstacles for the successful outcomes of immunotherapies [1,2].

The tumor microenvironment provides a milieu favoring the development of resistant mechanisms, which may preexist based on activating mutations in oncogenic pathways or can be generated as a feedback response to the intratumoral immune pressure. Such an acquired or adaptive immunoresistance is manifested primarily in the form of suppressor cells, inhibitory factors and inhibitory receptors and their ligands, all of which, alone or in combination, counteract tumor-directed immune reactivity in the form of reinforced endogenous antitumor immunity and/or induced antitumor immunity during or after immunotherapies and targeted therapies [2]. In this sense, immunoresistance could more accurately reflect the differential levels of immunosurveillance and, to a lesser extent, immune evasion. For instance, several reports have demonstrated the association of PD-L1 and other immune checkpoint ligands or their receptors with an abundance of immune infiltrates and Th1 cytokine secretion by these cells, reflecting the presence of antitumor immunity in the tumor compartments [3,4,5]. In addition, an increase in the density of FoxP3+ Tregs in the tumor tissue has been suggested to be a consequence of tumor-directed immune response, representing a contra regulatory response to the preexisting tumor immune aggression [6]. In the same line, higher protein levels of the immunosuppressive indoleamine 2,3-dioxygenase (IDO) at baseline were predicted to have favorable clinical responses in patients receiving immunotherapy based on the administration of ipilimumab [7,8,9]. Tumor-associated macrophages (TAMs) are the most abundant immune cells in many solid tumors, and TAM-infiltration strongly correlates with tumor progression and poor prognosis in various solid tumors [10,11,12,13,14] and lymphoma [15]. While macrophages retain phenotypic and functional plasticity, the majority of TAMs are immune suppressive, M2-like macrophages with complex pro-tumor functions. TAMs secrete various cytokines and growth factors, including interleukin (IL)-10, transforming growth factor-beta (TGF-β), vascular endothelial growth factor (VEGF), and C-X-C motif ligand (CXCL) 12 to drive cancer progression through immune suppression, tumor angiogenesis, invasion and metastasis [16,17,18]. TAMs also play critical roles in the response and resistance to common cancer therapies, such as chemotherapy, radiation therapy [19], angiogenesis [20], and hormone deprivation therapy [21], and numerous macrophage-modulating approaches have shown improved therapeutic efficacy in preclinical studies [22,23,24,25]. CD163 is often used as their specific marker [26,27].

Acquired or adaptive immunoresistance in the tumor tissue might represent a powerful biomarker for a robust local antitumor immune response. However, so far there is no study to show if, and to what extent, either type of immunoresistance can occur before any therapies as a consequence of a rise in immune infiltrates in the various tumor compartments, and whether this could function as a favorable prognostic biomarker. This is the first study to report a direct and strong correlation between densities of CD163+ or FoxP3+ cells and densities of CD8+ cells combined in the tumor center (TC) and tumor invasive margin (IM). A similar situation was also described when TC and IM were separately analyzed. Moreover, we provide data to show poor clinical outcomes in breast cancer patients with exceedingly high CD8+ TC/IM ratios, which were associated with increased frequencies of CD163+ and FoxP3+ cells. In contrast, patients with moderate or high CD8+ TC/IM ratios had decreased frequencies of CD163+ and FoxP3+ cells and better clinical outcomes. Thus, we may propose that the rise of suppressor immune phenotypes intratumorally represents a negative feedback response to a robust endogenous antitumor immunity.

## 2. Materials and Methods

### 2.1. Patient Selection and Characteristics

A total of 97 tissue samples were available from patients with histologically-confirmed invasive ductal BCa, without metastatic disease, diagnosed between 2000 and 2015. Clinical follow-up data were available for all 97 patients, with a median follow-up period of 6.88 years (range: 0.11–10 years). The study was approved by the Institutional Review Board of Saint Savas Cancer Hospital (IRB-ID 6079/448/10-6-13). The clinicopathological characteristics of the patients are presented in Table 1. 

### 2.2. Assessment of Tumor-Infiltrating Leukocytes 

**Figure 1:**
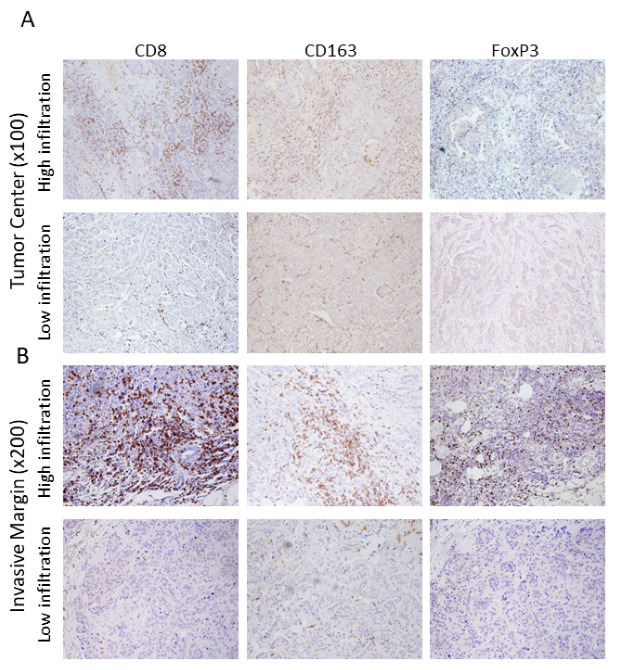
Immunohistochemically stained images of tumor compartments (**A**) Tumor Center (×100) and (**B**) Invasive Margin (×200) infiltrated with CD8+ and FoxP3+ cells and CD163+ macrophages.

Haematoxylin–Eosin-stained tumor slides were reviewed by two breast pathologists to select the most representative slide of each tumor. Formalin-fixed paraffin-embedded (FFPE) tissue blocks were obtained after surgery from the Saint Savas Pathology Department and analyzed for tumor immune cell infiltration, as described in detail in our previous reports [28,29]. Immunostaining, with CD8 (SP16, 1:80; Thermo Scientific, Waltham, MA, USA), CD163 (10D6, 1:400; Biocare) and FoxP3 (236A/E7, 1:100; Abcam), was performed for all patients using the Leica Bond III automation (Leica Biosystems, Nußloch, Germany) and Leica detection kit (Leica Biosystems, Newcastle, UK), as previously described. Quantification of the infiltrating cells/mm^2^ was performed, as described in [28,29,30]. Representative staining of the tumor samples for the CD8+, FoxP3+ and CD163+ cells in the tumor center and invasive margin are shown in Figure 1.

### 2.3. Statistical Analysis

The GraphPad Prism v.8.0 software was used for cumulative survival probabilities testing by Kaplan-Meier analysis, with 95% confidence intervals (95%-CIs), and comparison using log rank and Gehan Breslow tests. *p* values < 0.05 were considered statistically significant.

## 3. Results

### 3.1. Combined Intratumoral Analyses in TC and IM: Association between CD8+ and CD163+ or FoxP3+ Cell Densities

**Figure 2:**
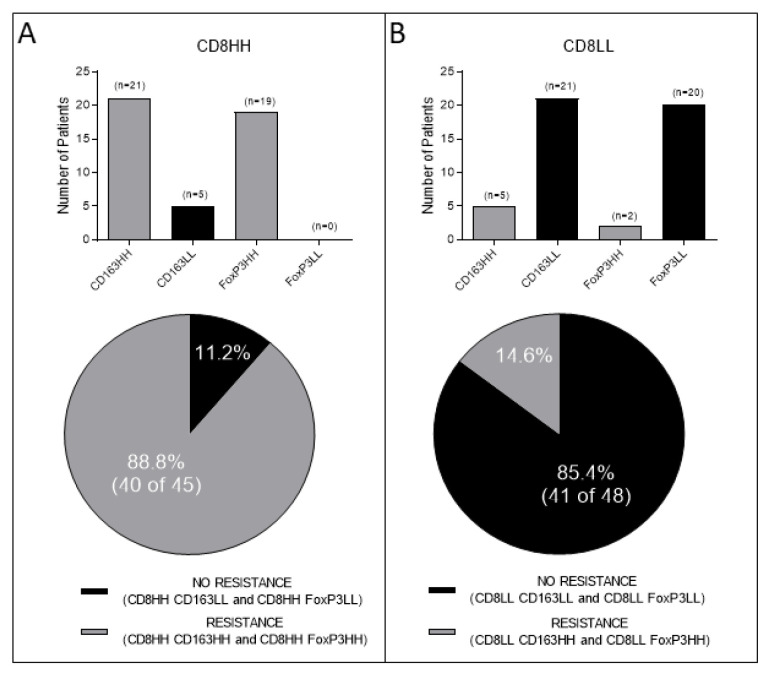
Association between CD8+ and CD163+ or FoxP3+ cell densities jointly in the Tumor Center (TC) and in the Invasive Margin (IM). (**A**) Number of patients with high CD8+ densities in the TC and IM (CD8HH) and either high or low densities of CD163+ (CD163HH or CD163LL, respectively) or high or low densities of FoxP3+ (FoxP3HH or FoxP3LL, respectively) cells. (**B**) Number of patients with low CD8+ (CD8LL) densities and either high or low densities of CD163+ or FoxP3+ cells in the TC and IM.

Initially, we estimated the absolute counts of CD8+, CD163+, and FoxP3+ cells jointly in the TC and IM. Patients’ tumors with high CD8+ cell densities in both, TC and IM (designated as CD8HH) (*n* = 45), in their vast majority also had high densities in both regions of either CD163+ cells or FoxP3+ cells (i.e., CD163HH; *n* = 21 of 45 or FoxP3HH; *n* = 19 of 45, respectively; total *n* = 40 of 45; 88.8%). Only a marginal number of patients in this group had tumors with low numbers of CD163+ cells (i.e., CD163LL: *n* = 5 of 45; 11.2%), whereas none had tumors with low FoxP3+ densities (i.e., FoxP3LL) (Figure 2A). Interestingly, tumors displaying low densities of CD8+ cells in both tumor regions (i.e., CD8LL; *n* = 48) were vastly co-infiltrated by low numbers of CD163+ or FoxP3+ cells (CD163LL: *n* = 21 of 48; FoxP3LL: *n* = 20 of 48; total: 41 of 48; 85.4%), whereas tumors displaying high densities of suppressor elements (i.e., either CD163HH or FoxP3HH) could be detected only in a restricted number of patients (*n*= 7 of 48; 14.6%) (Figure 2B).

### 3.2. Combined Intratumoral Analyses in TC and IM: Association between CD8+ and Combined CD163+, FoxP3+ Cell Densities

**Figure 3:**
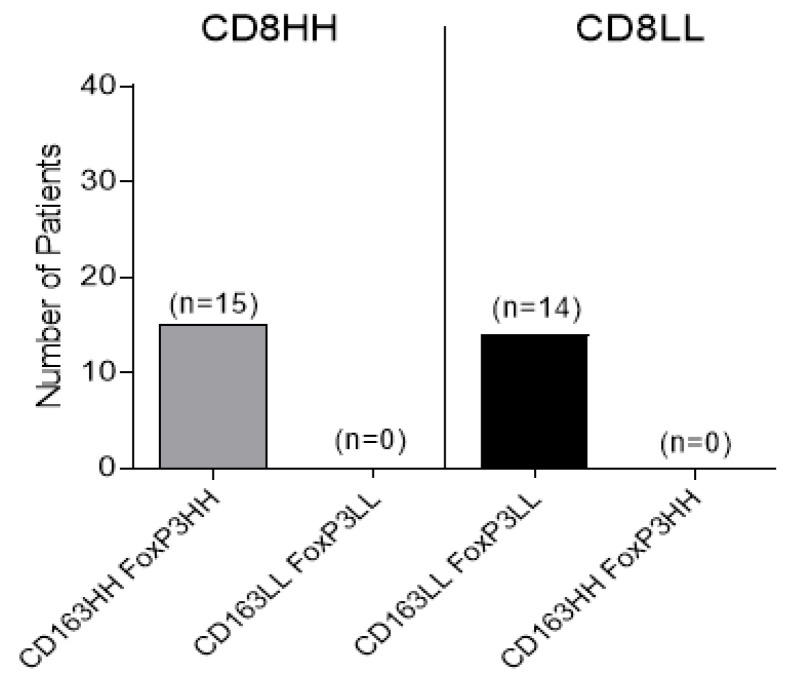
Association between CD8+ and combined CD163+, FoxP3+ cell densities in TC and IM. The number of patients with high densities of CD8+ cells (CD8HH) and densities of suppressive cell populations (CD163HH/FoxP3HH or CD163LL/FoxP3LL) are shown on the left panel. The number of patients with low densities of CD8+ cells (CD8LL) and densities of suppressive cell populations (CD163HH/FoxP3HH or CD163LL/FoxP3LL) are shown on the right panel.

Our results so far suggested that the infiltration of tumors jointly in the TC and IM by high densities of CD163+ or FoxP3+ cells occurs predominantly in cases where the same tumors are also co-infiltrated in these compartments by high densities of CD8+ cells. In the course of our studies, by analyzing the association between CD8+ cell frequencies with combined densities of CD163+ and FoxP3+ cells, we found a more profound expression of this infiltration profile. Thus, as shown in Figure 3, we could score 15 tumors in which the CD8HH were associated with CD163HH and FoxP3HH (15.5% of total studied samples), but not a single tumor with CD8HH co-infiltrated by CD163LL and FoxP3LL. Inversely, we had 14 tumors possessing CD8LL combined with CD163LL and FoxP3LL and none with CD163HH and FoxP3HH.

### 3.3. Analyses in the TC or IM Separately

**Figure 4:**
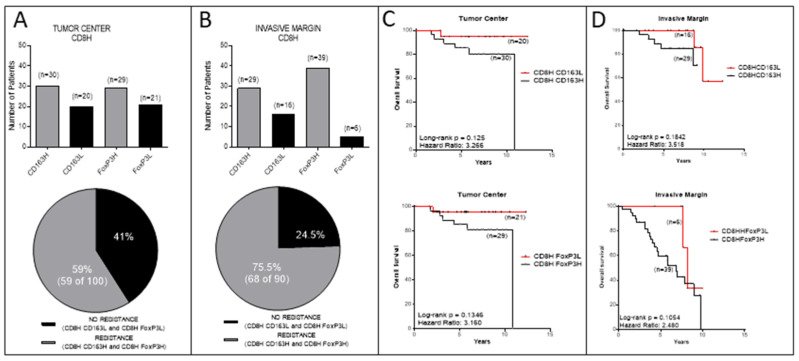
Association between CD8+ and CD163+ or FoxP3+ cell densities separately in the TC and in the IM and correlation with overall survival (OS). (**A**) Number of patients with high CD8+ cell densities in the TC (CD8H) and either high or low densities of CD163+ (CD163H or CD163L, respectively) or high or low densities of FoxP3+ (FoxP3H or FoxP3L, respectively) cells. (**B**) Number of patients with high CD8+ densities in the IM (CD8H) and either high or low densities of CD163+ (CD163H or CD163L, respectively) or high or low densities of FoxP3+ (FoxP3H or FoxP3L, respectively) cells. (**C**) Kaplan-Meier survival curves for patients with high densities of CD8+ cells and high or low densities of CD163+ cells (upper panel) and for patients with high densities of CD8+ cells and high or low densities of FoxP3+ cells (lower panel) in TC. (**D**) Kaplan-Meier survival curves for patients with high densities of CD8+ cells and high or low densities of CD163+ cells (upper panel) and for patients with high densities of CD8+ cells and high or low densities of FoxP3+ cells (lower panel) in IM.

Our analyses from the combined tumor regions (TC and IM) have demonstrated that the majority of patients’ tumors infiltrated by high numbers of CD8+ cells are also co-infiltrated by high densities of CD163+ and/or FoxP3+ cells. Next, we were keen to find out if an association between CD8H with CD163H and/or FoxP3H exists in each tumor region, separately. In addition, in this type of analyses our data showed a preferential association between high densities of CD8+ (CD8H) and CD163+ (CD163H) or FoxP3+ (FoxP3H) cells. As shown in Figure 4A, of the total patients having tumors with CD8H in TC, 59 patients had either CD163H (*n* = 30) or FoxP3H (*n* = 29). This preferential association of CD8H with either CD163H or FoxP3H was more profound in the IM (Figure 4B): 75.5% of patients with CD8H (*n* = 68 of total 90) had either CD163H (*n* = 29) or FoxP3H (*n* = 39). Only a minority of patients with CD8H in their tumors had either CD163L or FoxP3L in the TC or IM (in total, 41% and 24.5%, respectively) (Figure 4A,B). Furthermore, patients with tumors containing high densities of CD163+ or FoxP3+ cells in their TC had a worse prognosis compared to those with tumors infiltrated by these cell types at low densities, despite the fact that both groups of patients exhibited high levels of CD8+ in their tumors (Figure 4C). To this end, we found trends for improved overall survival (OS) in patients with CD8H and CD163L vs. those with CD8H and CD163H (Figure 4C, upper part), or in patients with CD8H and FoxP3L vs. those with CD8H and FoxP3H (Figure 4C, lower part), albeit by strong hazard ratios (HRs). Similarly, we also found trends with high HRs for favorable clinical outcomes when comparing the same groups in the IM (Figure 4B,D). The reason that may account for not obtaining statistical significance among these groups may relate to the limited number of patients examined. 

**Figure 5:**
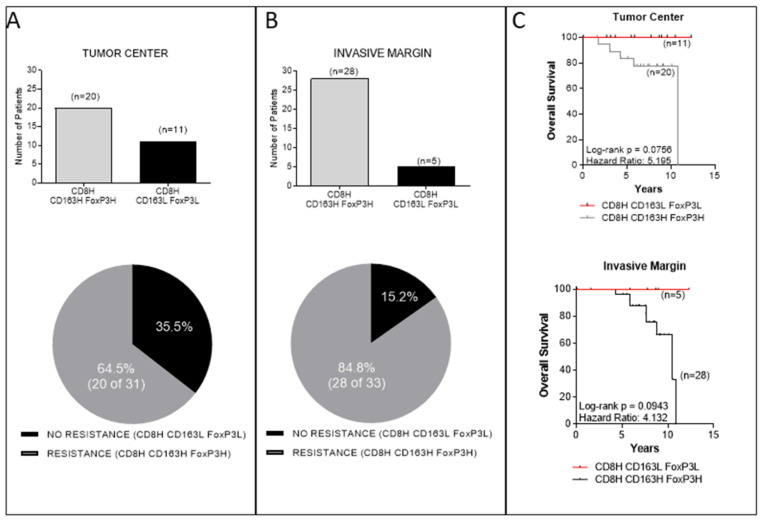
Association between combined densities of CD163+ and FoxP3+ cells in the context of high CD8+ densities separately in the TC and in the IM and correlation with overall survival (OS). Number of patients with high densities of CD8+, and high or low densities of CD163+ and FoxP3+ cells in TC (**A**) and in IM (**B**) and correlation with OS (**C**).

In the previous paragraph, we separately analyzed the densities of either CD163+ or FoxP3+ cells along with CD8H. Next, we estimated the association between combined densities of CD163+ and FoxP3+ cells in the context of CD8H. The data depicted in Figure 5A, B, show a preferential association between CD8H with CD163H and FoxP3H in the TC (CD8HCD163HFOXP3H) (20 of 31 patients; 64.5%), which was much more profound in the IM (28 of 33 patients; 84.8%). Only in the minority of cases were there associations between CD8H with CD163L and FoxP3L (CD8HCD163LFOXP3L) (35.5% and 15.2% in the TC and IM, respectively). OS analyses revealed a strong trend of increased OS with high HRs in favor of patients with high density of CD8+ cells and low combined densities of CD163+ and FoxP3+ cells (CD8HCD163LFOXP3L) vs. high density of CD8+ cells and high combined densities of CD163+ and FoxP3+ cells (CD8HCD163HFoxP3H) in the TC and the IM (Figure 5C). 

### 3.4. Correlations between CD8+ TC/IM Ratios and OS

**Figure 6:**
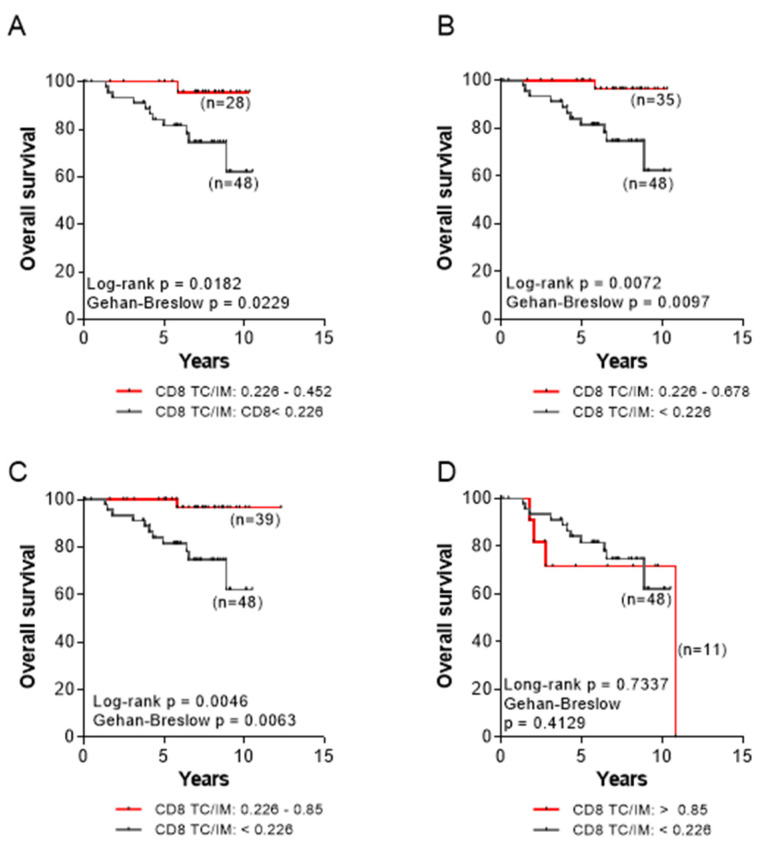
Correlations between CD8+ TC/IM ratios and overall survival for patients with (**A**) CD8+ TC/IM ratios between 0.226–0.452 and CD8+ TC/IM ratios below 0.226. (**B**) CD8+ TC/IM ratios between 0.226–0.678 and CD8+ TC/IM ratios below 0.226. (**C**) CD8+ TC/IM ratios between 0.226–0.85 and CD8+ TC/IM ratios below 0.226; and (**D**) CD8+ TC/IM ratios above 0.85 and CD8+ TC/IM ratios below 0.226.

The data so far suggested that high densities of CD8+ cells in TC or IM are associated with high densities of cells with suppressor phenotypes (i.e., CD163+ and FoxP3+) and unfavorable OS. Considering that interactions between tumor cells with immune elements intratumorally are dynamic with cells migrating from the IM to the TC, a process controlled by fibroblasts and chemokine gradients [31,32], we estimated the density relation of CD8+ cells in the TC and IM, best reflected by their TC to IM ratios, and looked for associations with OS. From our analyses, the median CD8+ TC/IM value was 0.226. Patients whose tumors were scored with CD8+ TC/IM values up to two-times higher than the median value (i.e., 0.452; Figure 6A), or up to three-times higher (0.678; Figure 6B) or up to four-times higher (with the highest value being 3.76-higher i.e., 0.85; Figure 6C) than the median value, had significantly higher OS compared to those with CD8+ TC/IM below the median value. Remarkably, patients with exceedingly high CD8+ TC/IM ratios (>0.85; range 3.8–53) (Figure 6D) had reduced OS, similar to that of patients with CD8+ TC/IM below the median value. 

**Figure 7:**
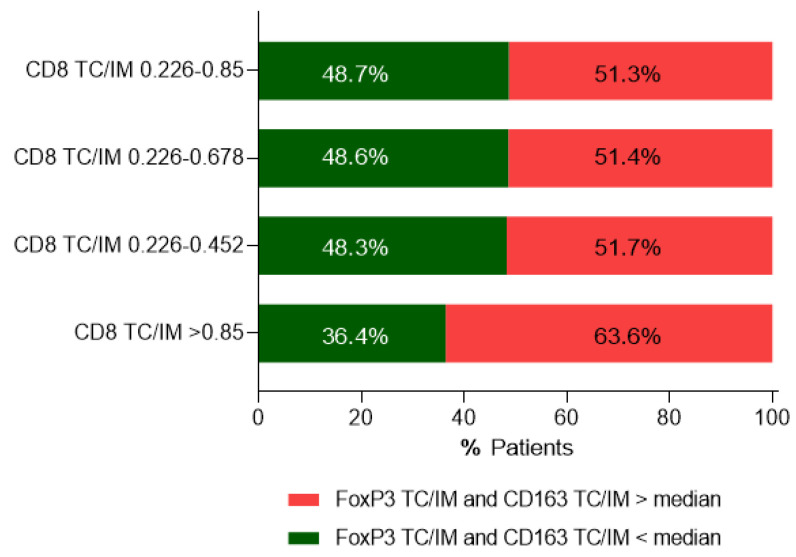
Correlations between CD8+ TC/IM ratios and CD163+TC/IM and FoxP3+ TC/IM ratios. For each CD8+ TC/IM ratio, the percentages of patients with CD163+ TC/IM and FoxP3+ TC/IM ratios above median are presented in red color. Accordingly, the percentages of patients with CD163+ TC/IM and FoxP3+ TC/IM ratios below median are presented in green color.

The majority of patients belonging to this latter group (63.6%; Figure 7) had their CD163+ TC/IM and FoxP3+ TC/IM ratios above median (median TC/IM values for CD163: 0.270 and for FoxP3: 0.341), ranging between 0.301–2.792 (1.1–10.34 higher) for CD163+ TC/IM and between 0.389–3.932 (1.1–11.53 higher) for FoxP3+ TC/IM ratios. 

**Figure 8:**
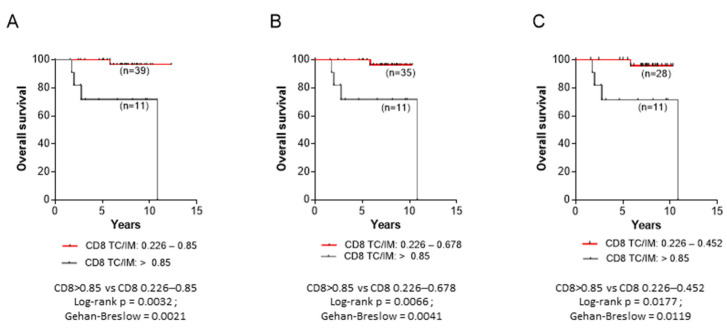
Correlations between CD8+ TC/IM ratios and overall survival for patients with (**A**) CD8+ TC/IM ratios between 0.226–0.85 and CD8+ TC/IM ratios above 0.85. (**B**) CD8+ TC/IM ratios between 0.226–0.678 and CD8+ TC/IM ratios above 0.85. (**C**) CD8+ TC/IM ratios between 0.226–0.452 and CD8+ TC/IM ratios above 0.85.

Additionally, we could also show significantly improved OS in patients with CD8+ TC/IM values up to 0.85; namely, 0.226–0.85, 0.226–0.678 and 0.226–0.452, as compared to those with CD8+ TC/IM values > 0.85 (Figure 8A, B and C, respectively).

## 4. Discussion

In this study, we evaluated the frequencies of FoxP3+ and CD163+ cells in the TC and IM in primary breast tumors and found an accumulation in either of these areas which was associated with increased densities of CD8+ cells. Analogous to this, tumor regions with low levels of CD8+ cells were characterized by low densities of FoxP3+ and CD163+ cells. We could demonstrate a direct correlation between the frequencies of CD8+ cells with CD163+ and/or FoxP3+ cells in single or combined tumor compartments (IM and/or TC) in the vast majority of patients’ tumors. This high vs. low frequency distribution of cells with apparent functionally opposite immune phenotypes (i.e., suppressor vs. adaptive) in the tumor regions suggests that their accumulation in the tumor is selective. Our study additionally proposes a possible role for tumors to attract FoxP3+, as well as CD163+ cells, that may afterwards participate in suppression pathways, resulting in the impairment of endogenous antitumor immune responses within the tumor microenvironment. Accordingly, their abundance in the primary tumor before the initiation of standard therapies may be the result of a preexisting and prolonged immunity specifically directed against an immunogenic tumor. In that case, immunologically “hot” tumors should be able to trigger an endogenous antitumor immune reactivity. On these grounds, we propose that the increased densities of FoxP3+ and/or CD163+ cells in the intratumoral sections may represent a potent immunologic cellular marker of a local preexisting antitumor response.

Tregs are considered to function as suppressors of the antitumor immune response via cell to cell interaction and/or via releasing suppressor enzymes [33]. Decreased densities of Tregs intratumorally resulted in the failure of immunotherapies in preclinical tumor models and clinical studies [34,35]. In general, Tregs are considered to be a part of the tumor extrinsic acquired immune resistance (AIR), which functions to down-regulate therapy-induced adaptive antitumor immunity. Previous studies have already suggested that FoxP3 expression accurately defines the population of TGFβ expressing Tregs that accumulate in tumor sites where antitumor immunity is evident [36,37]. Based on these reports, we believe that, in our case, FoxP3+ cell-infiltrates might be an indirect indicator of a preexisting antitumor immune response in breast tumors. The fact that we could detect Tregs intratumorally before therapy could eventually point to the presence of a preexisting and durable antitumor immune response, which presupposes that the tumor is immunogenic enough to trigger an endogenous immune response. 

CD163 is a cell-surface glycoprotein that characterizes tumor-associated M2 macrophages [38,39,40]. The accumulation of CD163+ macrophages intratumorally in dense frequencies is an unfavorable prognosticator in many types of cancer, including breast cancer [38,41]. In our recent study [29], we showed that high frequencies of CD163+ cells combined with low frequencies of CD8+ cells in the center of breast tumors represents a significant biomarker for poor prognosis. In this case, in which way do Tregs and CD163+ macrophages migrate to the tumor? Reports from various studies have shown that a network consisting of multiple chemokine receptors expressed in combination, along with their specific chemokine ligands, plays a significant role in the trafficking of both FoxP3+ and CD163+ cells into the tumors, thus providing a mechanistic explanation for their role in regulating preexisting antitumor responses [32,42,43,44].

Our data also show that patients with high densities of suppressor FoxP3+ or CD163+ cells in their tumor center or the invasive margin, albeit with high numbers of CD8+ cells, had trends for shorter OS compared to those who displayed low FoxP3+ or CD163+ cell frequencies (Figure 4). Statistical significance was not reached, most likely due to the rather short clinical follow-up (median 6.88 years), as well as the relatively low number of patients per group. However, when densities of FoxP3+ and CD163+ cells were jointly considered, either in the tumor center or the invasive margin (Figure 5), the statistical difference was improved. This could be explained through a robust suppression of the antitumor immune response, when both suppressor cell-types are in high numbers intratumorally, which further decreases patients’ OS. It is also intriguing that in the group of patients with low FoxP3+ and/or CD163+ cell densities, the hazard ratios were high (between 2.480 and 5.195), meaning that these patients had a much lower risk of death. Another interesting finding from our studies was the decreased OS in patients with exceedingly high CD8+ TC/IM ratios. Tumors from these patients also had high FoxP3+ and CD163+ TC/IM ratios. Thus, we may propose that the short OS in these patients was due to an AIR, developed by the tumors via the recruitment of FoxP3+ and CD163+ suppressors in order to counteract the robust endogenous antitumor immunity. On these bases, we would like to propose the hypothetical scenario that the FoxP3+ and CD163+ cells which infiltrate the tumor constitute suppressor circuits, which are, in essence, derived by and are intrinsically linked to the immune system, rather than being organized by the tumor cells. This, in turn, suggests that patients with T-cell inflamed tumors, as a result of robust preexisting antitumor immunity, might be amenable to therapeutic treatments aiming at reversing regulatory immune pathways. In that case, CD163+ and FoxP3+ cells might function as immune-based negative feedback mechanisms, following the initial accumulation of CD8+ effector cells, which orchestrate the antitumor preexisting immunity intratumorally. A second hypothesis would be that a greater Treg/CD163 expression may be due to the expression of chemotactic cytokines or chemokines, or both, to attract these cells into the tumor, thus shaping a more favorable cancer microenvironment. 

The high density of preexisting immune effector cells at the site of the tumor is suggestive of a specific immune response to tumor antigens, despite the fact that these cells are restrained functionally once they enter the tumor [45]. Nonetheless, by reversing the intratumoral negative regulatory mechanisms, it is possible to reinvigorate preexisting immunity, thus providing clinical benefits [46]. Given the functional plasticity of T cells and macrophages during the development of antitumor immune responses [47], it is often the case that antitumor signaling processes can ultimately induce feedback inhibition, compromising the reactivity against the tumor [48]. Tumor adaptation takes advantage of this delicate balance of positive and negative immune signaling factors, allowing the cancer to generate resistance and progress [2]. Thus, a dynamic interaction between these elements of the adaptive immunity and tumor cells will lead to the generation of memory, which is tightly linked to the efficacy of immunotherapy. However, in the presence of robust immune suppression beyond the PD-1/PD-L1 axis, immunotherapies will ultimately fail, making it necessary to therapeutically target such suppressor pathways. 

Moreover, in patients with CD8+ high intratumoral frequencies, we could demonstrate that high CD8+ TC/IM density ratios were correlated with improved OS, only when these were fairly above the median CD8+ TC/IM ratio (i.e., up to almost 4-fold). Interestingly, we found that patients with exceedingly high CD8+ TC/IM ratios (reaching even 53-fold increases above median) had OS comparable to that observed in patients with low CD8+ TC/IM ratios (i.e., below median). In addition, the majority of patients who exhibited extremely high CD8+ TC/IM ratios also had high CD163+ and FoxP3+ TC/IM density ratios (up to 10-fold above median). This finding suggests that the tumors, when under robust immune pressure, fight back by recruiting suppressor elements into their microenvironment. Such a tumor extrinsic AIR to counteract the endogenous antitumor immunity could also take place during immunotherapies, thus generating robust intratumor antitumor responses. To this end, combinations of immune checkpoint inhibitors with drugs targeting intratumoral immune suppression mediated by FoxP3+ cells or macrophages [49,50,51,52] would be needed for improving clinical outcomes.

There are limitations in this work. One limitation is that it includes retrospectively collected cases; therefore, there is an absolute need to validate these data in large prospective studies. Another weakness is that, in our combined intratumoral analyses in TC and IM (Figure 2), we could not make statistical correlations for clinical outcomes between groups of patients whose tumors had high vs. low CD163+ or FoxP3+ cell infiltration combined with CD8HH (i.e., CD8HHCD163HH vs. CD8HHCD163LL or CD8HHFoxP3HH vs. CD8HHFoxP3LL), due to the scarcity of cases with CD8HH and CD163LL or FoxP3LL infiltrates. Another limitation is that our method is unable to characterize the function of the analyzed cell infiltrates in the tumor compartments.

Several approaches targeting CD163+ pro-tumor macrophages and/or immune suppressive FoxP3+ have been associated with an improved response to immunotherapy and/or chemotherapy and are in pre-clinical or clinical development [53,54,55,56,57,58,59,60]. Our data, while preliminary, would support combined regimens of immunotherapy or chemotherapy with the addition of FoxP3+ and CD163+ macrophage-targeting agents in breast cancer.

## 5. Conclusions

In this report, we have shown a direct correlation between densities of CD8+ cells with densities of CD163+ and FoxP3+ cells in the primary tumors of patients with breast cancer. In the majority of the cases analyzed, we found that cells with suppressor phenotypes (i.e., CD163+ and FoxP3+ cells) are abundant in the tumor, only in the presence of high frequencies of cells with cytotoxic effector phenotypes (i.e., CD8+ cells). This is suggestive of an immune-intrinsic negative feedback mechanism of AIR, which is developed by a robust endogenous antitumor immune response.

## Figures and Tables

**Table 1:** Clinicopathological characteristics of patients.

Total Number of Patients	
*n* = 97	
Median age (years)	Range
53	32–78
Tumor size	*n*
Tx	1
T1	39
T2	50
T3	7
LN status	*n*
N0	38
N1	34
N2	18
N3	7
AJCC stage (TNM)	*n*
I	25
IIA	25
IIB	20
IIIA	19
IIIB	X *
IIIC	8
Grade	*n*
1	3
2	56
3	38
Hormone receptor	*n*
positive	74
negative	23
HER-2/neu	*n*
positive	23
negative	74

* Stage IIIB patients were not eligible.

## Data Availability

The data presented in this study are available upon reasonable request.
